# Haemostatic agents in apical surgery. A systematic review

**DOI:** 10.4317/medoral.21109

**Published:** 2016-07-31

**Authors:** Adrià Clé-Ovejero, Eduard Valmaseda-Castellón

**Affiliations:** 1 DDS. Resident of the Master of Oral Surgery and Implantology, University of Barcelona Dental School; 2 DDS, PhD, MS, EBOS. Professor of Oral Surgery, Professor of the Master of Oral Surgery and Implantology, Barcelona University Dental School. Researcher of the IDIBELL Institute

## Abstract

**Background:**

Blood presence in apical surgery can prevent the correct vision of the surgical field, change the physical properties of filling materials and reduce their sealing ability.

**Objetive:**

To describe which are the most effective and safest haemostatic agents to control bleeding in patients undergoing apical surgery.

**Material and Methods:**

TWe carried out a systematic review, using Medline and Cochrane Library databases, of human clinical studies published in the last 10 years.

**Results:**

The agents that proved more effective in bleeding control were calcium sulphate (100%) and collagen plus epinephrine (92.9%) followed by ferric sulphate (60%), gauze packing (30%) and collagen (16.7%). When using aluminium chloride (Expasyl®), over 90% of the apical lesions improved, but this agent seemed to increase swelling. Epinephrine with collagen did not significantly raise either blood pressure or heart rate.

**Conclusions:**

Despite the use of several haemostatic materials in apical surgery, there is little evidence on their effectiveness and safety. The most effective haemostatic agents were calcium sulphate and epinephrine plus collagen. Epinephrine plus collagen did not seem to significantly raise blood pressure or heart rate during surgery. Aluminium chloride did not increase postoperative pain but could slightly increase postoperative swelling. Randomized clinical trials are needed to assess the haemostatic effectiveness and adverse effects of haemostatic materials in apical surgery.

**Key words:**Haemostasis, apical surgery.

## Introduction

Apical surgery is a procedure performed to remove lesions around the apex of a tooth with the main aim of preserving it. This surgery is recommended (a) when radiological findings of apical pathology are detected, (b) when extruded material is observed in a tooth with clinical or radiological findings of apical periodontitis, (c) in persistent apical pathology when endodontic treatment is not possible and (d) in root perforation impossible to treat through a coronal access (1).

An excessive bleeding in oral surgery can increase the operation time and compromise the correct vision of the surgical field and hence the success of the treatment (2). The first step to prevent bleeding is a good assessment of bleeding risk. However, in apical surgery, a low profuse bleeding, typical of a healthy patient, may be enough to affect the sealing ability of filling materials (3-5). There are different haemostatic techniques or materials to reduce bleeding. Some of these proposed agents to control bleeding in apical surgery are: bone wax (6-8), cellulose membranes (9-11), gelatine sponges (10,11), collagen agents (9,11), thrombin (10-12), chitosan (9,13), epinephrine (11,14,15), ferric sulphate (16-21), calcium sulphate (16) and aluminium chloride (14,15,18-21). Different articles have addressed haemostatic agents for apical surgery, but to date there isn’t any specific systematic review on this topic.

The main objective of this review was defined using a PICO question format: to determine which is the most effective and safest haemostatic agent that can be used to control bleeding in patients undergoing apical surgery.

## Material and Methods

A systematic review was carried out and the PRISMA statement was followed. Medline (PubMed) and Cochrane Library databases were searched between November 2014 and February 2015. The inclusion criteria were: all human clinical trials regarding the effectiveness and/or adverse effects of haemostatic agents in apical surgery, with statistical data and published in the last 10 years in English, Spanish, French or German. The medical subject headings (MeSH) used in the Medline search were: Periapical Tissue/surgery; Blood Loss, Surgical/prevention and control; Oral Hemorrhage/prevention and control; Apicoectomy/methods; Hemostasis, Surgical/methods; Retrograde Obturation/adverse effects; Retrograde Obturation/methods; Retrograde Obturation/therapeutic use; Ferric Compounds/therapeutic use; Electrocoagulation/therapeutic use; Tampons, Surgical/therapeutic use; Aluminium Compounds/therapeutic use; Astringents/therapeutic use; Chlorides/therapeutic use; Calcium Sulphate/therapeutic use; Hemostatics/adverse effects; Hemostatics/therapeutic use; Alveolectomy/methods; Vasoconstrictor Agents/therapeutic use; Tooth Root/surgery. The terms used in the Cochrane Library search were Periapical Surgery Hemostasis and Endodontic Surgery Hemostasis. Additional articles were hand searched in the following journals: Restorative Dentistry & Endodontics, Dental Clinics of North America, Journal of Endodontics and Medicina Oral Patología Oral y Cirugía Bucal.

After eliminating duplicated articles, we discarded the irrelevant ones and only included clinical trials with statistical data in the qualitative synthesis of the systematic review. The list of references was searched to detect additional relevant articles. The studies were stratified using the Strength of Recommendation Taxonomy (SORT) scale according to their quality and consistency. Then, strength of recommendation level was given to the haemostatic agents.

The variables analysed were two: the effectiveness and the adverse effects of the haemostatic agents. The effectiveness was obtained from the articles, which subjectively assessed bleeding. It was recorded as the percentage of cases where a correct haemostasis was achieved. Adverse effects were rated according to each agent (visual analogue scale for pain and swelling, mmHg for blood pressure, beats/minute for heart rate and percentage for the failure rate). In addition, sample size, statistical tests and follow up were recorded from each article.

## Results

**Figure 1:**
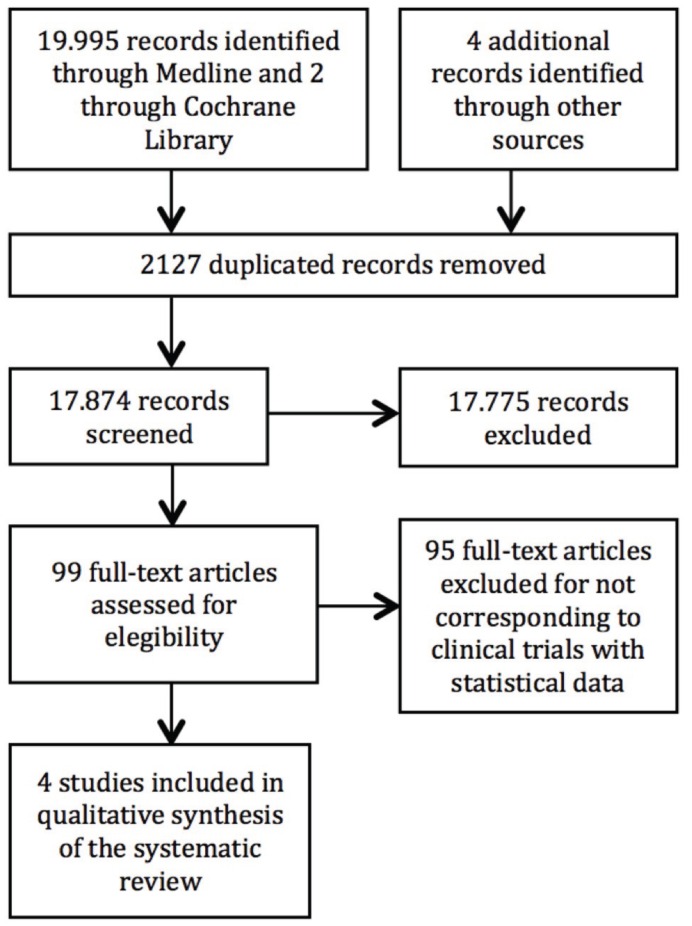
Flow Diagram (Prisma).

At the beginning, we identified 20.001 citations from the scientific literature search (19.995 from Medline, 2 from Cochrane Library and 4 additional articles). Duplicates were eliminated (2.127) and irrelevant articles were discarded (17.775). Ninety-nine articles were fully read. Only those corresponding to clinical trials with statistical data were selected. Finally, 4 articles were included in the qualitative synthesis of the systematic review (Fig. 1).

These 4 articles were classified according to the SORT scale. All of them were consistent, had a 2 level quality and the strength of recommendation in favour of the haemostatic techniques was B ( Table 1). The main results of the four clinical trials are described and summarized in  Table 2.

**Table 1:**
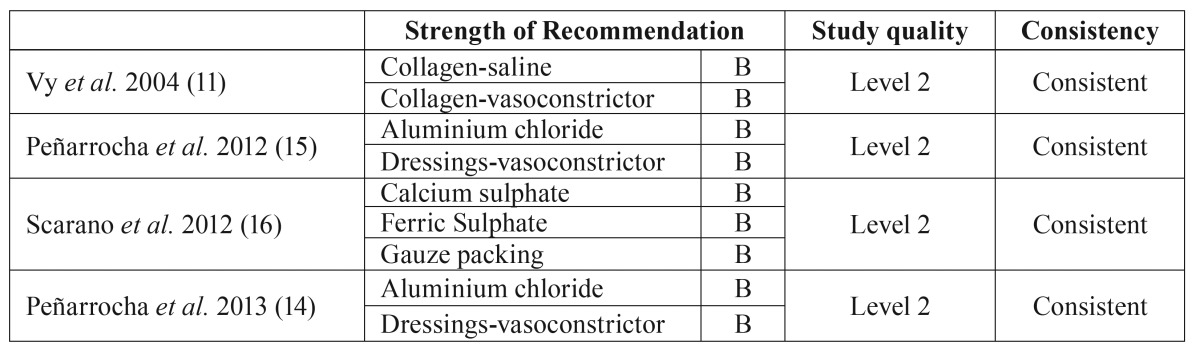
SORT scale. Scientific evidence level.

**Table 2:**
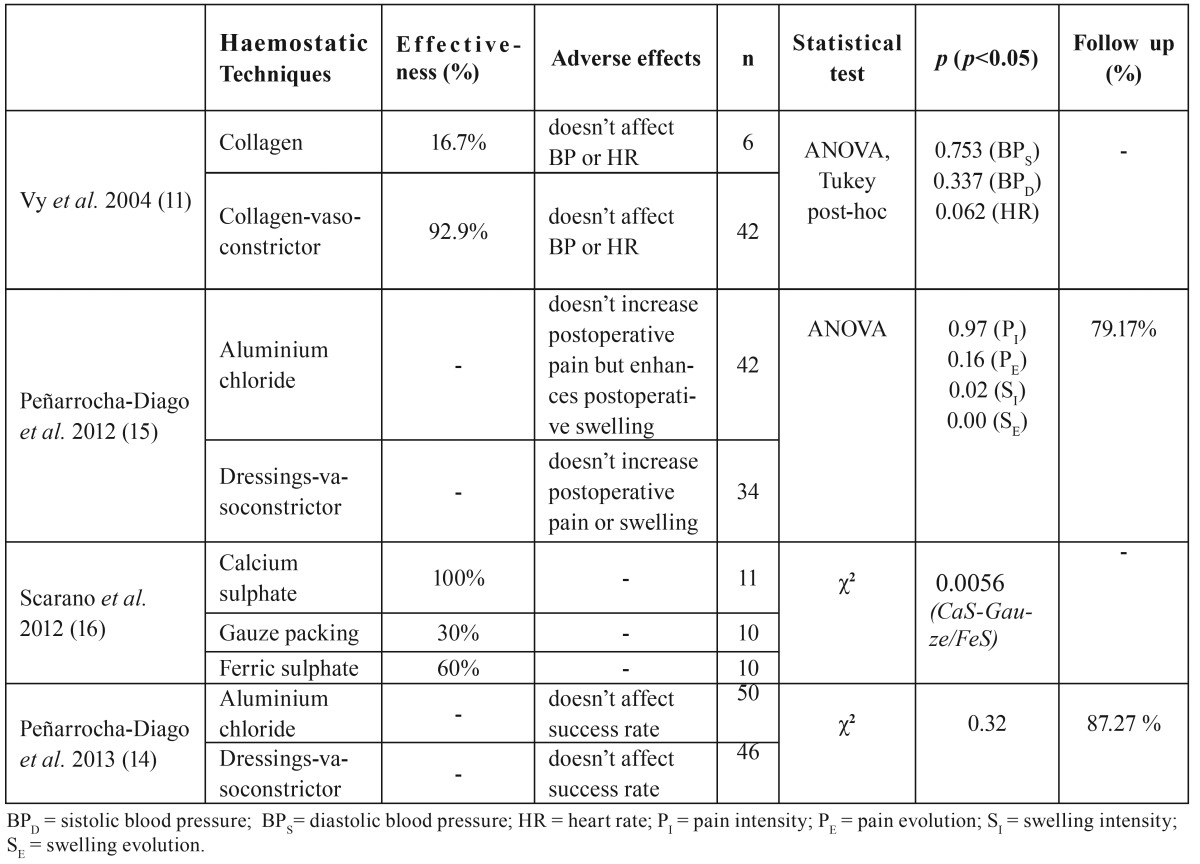
Main results.

The only study that analysed both the effectiveness and the adverse effects of haemostatic agents used to control bleeding in apical surgery was written by Vy *et al.* (11). This prospective study (level of evidence-2) compared the haemostatic effectiveness of Colla Cote® (collagen sponges) plus saline solution with Colla Cote® saturated with 2.25% racemic epinephrine. In addition, patients’ heart rate and blood pressure were monitored preoperatively and during anesthesia, surgery and postoperatively to detect differences between vasoconstrictor group (experimental group) and saline group (control group). In the 83.3% (5/6) of the cases where collagen-saline was used, a correct haemostasis couldn’t be achieved and other techniques had to be used to stop bleeding. In the collagen-vasoconstrictor group 93% (39/42) of the cases reached an ideal haemostasis, while in the 5% (2/42) there was mild and intermittent bleeding that did not jeopardize the surgical procedure. Only in a 2% (1/42) bleeding remained the same. Systolic blood pressure, diastolic blood pressure and heart rate were not significantly different between groups.

In the study of Peñarrocha-Diago *et al.* (15) (level of evidence 2) neither Expasyl® (aluminium chloride) or sterile dressings impregnated with local anesthetic solution with epinephrine (articaine 4% plus epinephrine 1:100.000) as haemostatic agent increased postoperative pain and swelling after apical surgery. Pain (as measured in a visual analogue scale) was found to be maximal 2 hours after surgery, coinciding with the end of the anesthetic effect. There were no statistically significant differences either in postoperative pain intensity or evolution. However, perceived swelling was more marked in the aluminium chloride group. (3.6 vs 5.5 mm), although swelling evolution followed similar patterns in both groups the first 12 hours. The aluminium chloride group had a further growth in the degree of swelling and both groups reached their highest peak at 48 hours. Thereafter, postoperative swelling in the aluminium chloride group decreased faster than the vasoconstrictor group until day 5, when differences began to reduce. Swelling was always higher in the aluminium chloride group. The data was compared with other studies that analysed postoperative pain and swelling after apical surgery and results were similar.

In the same year, Scarano *et al.* (16) published an experimental study (level of evidence-2) on the haemostatic effectiveness in apical surgery using calcium sulphate, ferric sulphate and gauze packing. In the calcium sulphate group, all operations (11/11) were carried out under a suitable haemostatic control. In the ferric sulphate group, adequate haemostasis was achieved in 60% of the cases (6/10) and in the group of gauze packing, only 30% (3/10) of the cases were classified as adequate. The statistical analysis determined that there were only statistically significant differences between the calcium sulphate and the other two techniques (*p*=0.0056).

In 2013, Peñarrocha-Diago *et al.* (14) carried out another study on the success rate of apical surgery using Expasyl® (aluminium chloride) or sterile dressings impregnated with local anesthetic solution with epinephrine (articaine 4% plus epinephrine 1:100.000). With a follow-up of at least one year after surgery, no significant differences were detected between the two groups (*p*=0.32). Sixty-three per cent of the teeth were considered successful in the aluminium chloride group, while 31% improved and 6% failed. In the vasoconstrictor group 59% of the cases were classified as success, 28% improved and 14% failed.

## Discussion

The agents that have proved more effective for bleeding control are calcium sulphate (100%) and collagen-epinephrine (92.9%) followed by ferric sulphate (60%), gauze packing (30%) and collagen-saline (16.7%). One of the major limitations of these studies is that are not randomized (14,15) or it is not specified if they are (11,16,). Another important factor to take into account is the small sample size of Scarano *et al.* (16) which can lead to nonspecific results. In this study, the haemostatic effectiveness of ferric sulphate (60%) differs a little bit from a randomized study of Vickers *et al.* (22) in 2002 that showed a 94% (15/16) of adequate haemostasis using the same criteria to evaluate bleeding. Randomized studies with a larger sample are needed to endorse the effectiveness of calcium sulphate, quantify its differences with the ferric sulphate and find out its effect upon postoperative swelling risk. The fact that the criteria used to evaluate the haemostatic effectiveness are different among the studies makes comparison difficult. Scarano *et al.* (16) classified haemostasis as “adequate” when the root-end preparation was dry and haemorrhage-free during root-end filling procedures and “inadequate” when the root-end preparation was not either dry or haemorrhage-free. In contrast with Vy *et al.* (11) who used an index where “0” corresponded to a lack of haemorrhage control that compromised vision at the surgical site, “1” meant slight but apparent intermittent bleeding that persisted after the application of the sponges and “2” was identified as a complete haemorrhage control that provided a dry surgical field. To compare results of both studies and calculate the haemostatic success percentage of each technique, only cases identified as “adequate” or “grade 2” were taken into account. Consensus on an index or scale to evaluate the haemostatic effectiveness in apical surgery is needed to compare future results.

In regard to the adverse effects, neither epinephrine nor aluminium chloride seemed to interfere with the success of the surgery or cause any increase in postoperative pain. However, in the study of Peñarrocha-Diago *et al.* (15), the use of aluminium chloride caused a slight increase in postoperative swelling compared with the epinephrine. The authors attribute this swelling to the fact that the study was not randomised and not to any inflammatory reaction of Expasyl®. Indeed, an animal study of von Arx *et al.* in 2006 stated that this inflammatory reactions are limited to bone defects and no adverse tissue reactions are seen in the vicinity of bone defects (23). Future randomised studies in humans are needed to analyse this possible association.

Many publications have mentioned the importance of limiting the epinephrine doses in dentistry due to adverse effects (24,10). In the experimental group of Vy *et al.* (11), collagen sponges saturated with 10 drops of 2.25% racemic epinephrine were used as haemostatics. Therefore, the range of epinephrine used in this group was 2.1 to 7.5 mg, considering that each drop contained 0.21-0.25 mg of epinephrine and the maximum number of bony crypts per patient was 3. During monitoring no significant changes in heart rate neither blood pressure had been observed. The average values were 131/80 mmHg and 76 beats/minute in the collagen-saline group and 130/77 mmHg and 72 beats/minute in the collagen-vasoconstrictor group. Thus, collagen sponges together with this amount of racemic epinephrine was not only effective to control bleeding but also did not alter either blood pressure or heart rate during apical surgery. However, the time elapsed from the application of epinephrine until the end of the surgery was not specified. Future studies should assess if collagen or sterile dressings together with epinephrine from a local anesthetic solution also keeps blood pressure and heart rate at baseline values when used as haemostatics in apical surgery.

Randomized clinical trials are needed to increase the strength of recommendation of haemostatic agents in apical surgery and consensus criteria for assessing effectiveness and adverse effects should be adopted. Samples should also be large enough to study both haemostatic effectiveness and adverse effects.

## Conclusions

Several haemostatic materials and techniques for bleeding control during apical surgery have been described but without randomized clinical trials demonstrating their effectiveness. The limited quality and small number of the existing clinical trials must be taken into account.

• The most effective haemostatic agents are calcium sulphate and epinephrine plus collagen. The use of the last one did not increase either blood pressure or heart rate during surgery.

• Aluminium chloride did not increase postoperative pain but might slightly increase postoperative swelling. There is no evidence regarding its effectiveness.

• Ferric sulphate to control bleeding seems to have very limited evidence. Postoperative swelling might be a drawback.

• The use of collagen-saline and gauze packing without haemostatic solution has a low efficiency for bleeding control in apical surgery.
